# The influence of rising tropospheric carbon dioxide and ozone on plant productivity

**DOI:** 10.1111/plb.12973

**Published:** 2019-03-04

**Authors:** E. A. Ainsworth, P. Lemonnier, J. M. Wedow

**Affiliations:** ^1^ United States Department of Agriculture (USDA) Agricultural Research Service (ARS) Global Change and Photosynthesis Research Unit Urbana IL USA; ^2^ Department of Plant Biology and Institute for Genomic Biology University of Illinois at Urbana‐Champaign Urbana IL USA

**Keywords:** air pollution, CO_2_, Free Air CO_2_ Enrichment, global plant productivity, O_3_

## Abstract

Human activities result in a wide array of pollutants being released to the atmosphere. A number of these pollutants have direct effects on plants, including carbon dioxide (CO_2_), which is the substrate for photosynthesis, and ozone (O_3_), a damaging oxidant. How plants respond to changes in these atmospheric air pollutants, both directly and indirectly, feeds back on atmospheric composition and climate, global net primary productivity and ecosystem service provisioning. Here we discuss the past, current and future trends in emissions of CO_2_ and O_3_ and synthesise the current atmospheric CO_2_ and O_3_ budgets, describing the important role of vegetation in determining the atmospheric burden of those pollutants. While increased atmospheric CO_2_ concentration over the past 150 years has been accompanied by greater CO_2_ assimilation and storage in terrestrial ecosystems, there is evidence that rising temperatures and increased drought stress may limit the ability of future terrestrial ecosystems to buffer against atmospheric emissions. Long‐term Free Air CO_2_ or O_3_ Enrichment (FACE) experiments provide critical experimentation about the effects of future CO_2_ and O_3_ on ecosystems, and highlight the important interactive effects of temperature, nutrients and water supply in determining ecosystem responses to air pollution. Long‐term experimentation in both natural and cropping systems is needed to provide critical empirical data for modelling the effects of air pollutants on plant productivity in the decades to come.

## Past, Present and Future Trends in Tropospheric [CO_2_] and [O_3_]

**Figure 1:**
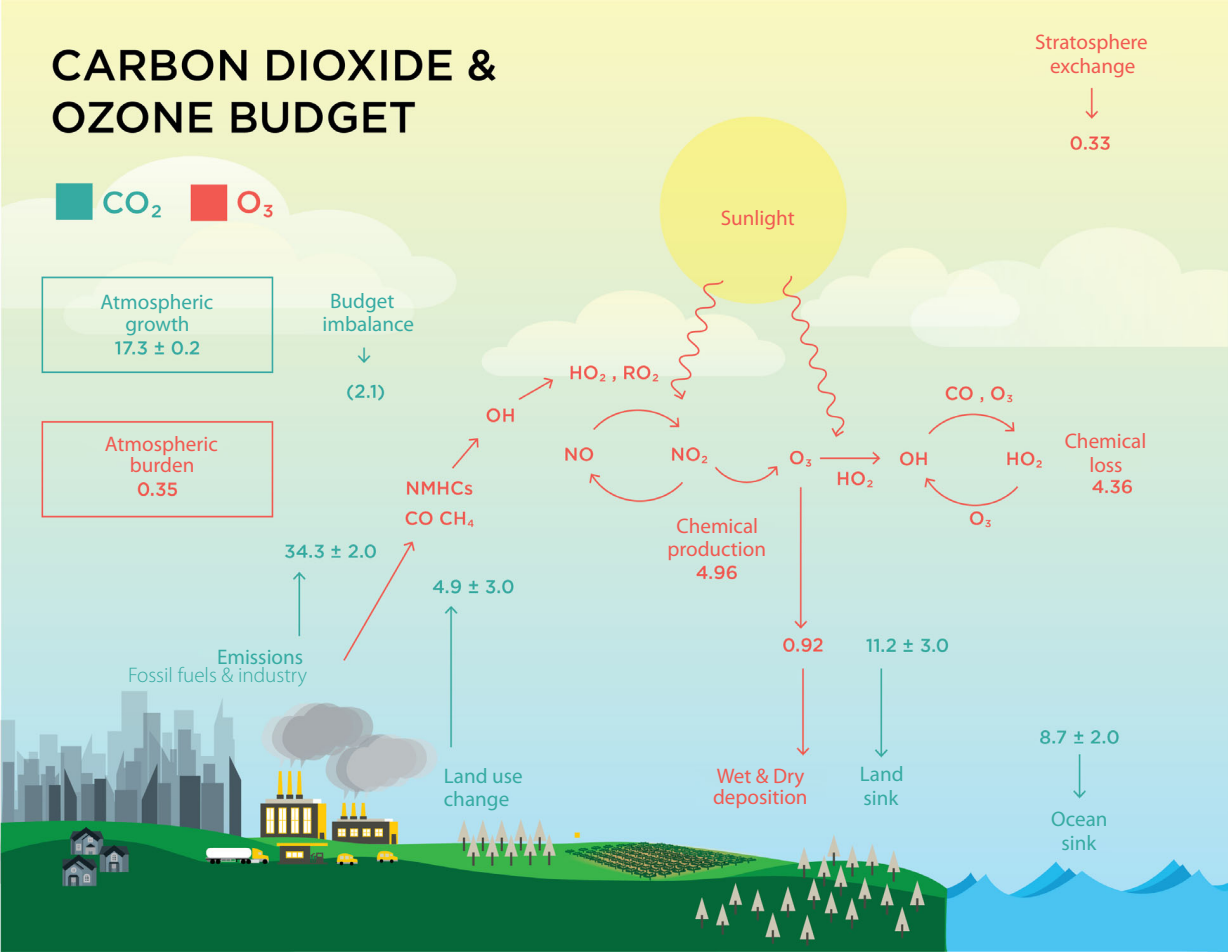
Schematic of the current global carbon dioxide and ozone budgets (Pg CO_2_ or O_3_). Pools and fluxes of CO_2_ (in green) are taken from the Global Carbon Project (Le Quéré *et al*. 2018). Pools and fluxes of O_3_ (in red) are taken from Hu *et al*. (2017).

Anthropogenic activities have been affecting the atmospheric concentration of greenhouse gases (GHG) arguably over the past 8,000 years, but most profoundly since the Industrial Revolution. Today, our atmosphere has the highest atmospheric concentrations of CO_2_ ([CO_2_]), methane (CH_4_), nitrous oxide (N_2_O) and tropospheric ozone ([O_3_]) experienced in the past 650,000 years, and these GHG are the main drivers of global warming observed since the mid‐20th century. Cumulative anthropogenic emissions of CO_2_ (2,040 Pg between 1750 and 2011) are dominantly responsible for global warming and can be divided in two main sources: land‐use change and fossil fuel consumption (Fig. 1). Until 1950, land‐use change was the major source of CO_2_ emissions but since then fossil fuels have dominated and now account for 88% of CO_2_ emissions (34.3 Pg CO_2_·year^−1^). Over the second half of the 20th century, fossil fuel and industrial CO_2_ emissions rapidly increased with an average growth rate of 1.1% per year in the 1990s and 3.3% per year in the 2000s. There are three major sinks for CO_2_ emissions: oceans, land and the atmosphere (Fig. 1). The strength of the ocean and land sinks discount the burden of CO_2_ remaining in the atmosphere. The land C sink has been increasing with growing fossil fuel CO_2_ emissions. From 1960 to 1979, the land CO_2_ sink was ~6.3 Pg CO_2_·year^−1^ and it grew to 9.8 Pg CO_2_·year^−1^ from 1996 to 2015 (Peñuelas *et al*. 2017) and to 11.2 Pg CO_2_·year^−1^ from 2007 to 2016 (Le Quéré *et al*. 2018; Fig. 1). Emissions stabilised for three consecutive years between 2014 and 2016, mainly attributable to decreased coal use in China and the United States, combined with improvements in energy efficiency and increased use of renewable energies (Jackson *et al*. 2017). Despite this hiatus, the gross world product (GWP) continued to increase, showing a global decoupling of these two parameters. Unfortunately, emissions continued on an upward trajectory again in 2017, with a 2% projected global growth rate of CO_2_ emissions from fossil fuels and industry mostly due to regional increases (3.5% in China and 2.0% in India). Future CO_2_ emissions over the 21st century will vary with socio‐economic development, population growth and climate policy, as explored by the Intergovernmental Panel on Climate Change (IPCC). To limit global warming to 2 °C above pre‐industrial levels, which is the target of the 2015 UN Paris Agreement, either rapid development and adoption of negative emission technologies is required, or total global emissions should not exceed 1000 Pg of CO_2_ from 2011 to 2100 (Anderson 2015).

Tropospheric ozone (O_3_) is a secondary pollutant formed as a by‐product of the photochemical oxidation of NO_x_ in the presence of carbon monoxide (CO), methane (CH_4_) and non‐methane hydrocarbons (Myhre *et al*. 2013; Fig. 1). The increased amount of tropospheric O_3_ from 1750 to 2011 has contributed to a radiative forcing of +0.40 W·m^−2^, making O_3_ the third most potent anthropogenic greenhouse gas following CO_2_ and CH_4_ (Myhre *et al*. 2013). It is estimated that global production of tropospheric O_3_ is ~5.29 Pg·year^−1^, produced primarily from chemical production (4.96 Pg·year^−1^) with a smaller contribution from stratospheric exchange (0.33 Pg·year^−1^; Hu *et al*. 2017). Chemical loss of O_3_ accounts for most of the O_3_ destruction in the troposphere, but dry deposition of O_3_ to the terrestrial surface is also a significant sink for O_3_, accounting for ~18% of removal from the troposphere (Hardacre *et al*. 2015; Fig. 1). While Fig. 1 shows the global average O_3_ budget, surface O_3_ concentrations are highly dynamic, and depend on temporal, seasonal and spatial factors. Concentrations typically peak in the late afternoon, with high [NO_x_] and sunlight levels, and are seasonally variable with low concentrations in cold winter months and high concentrations in warm summer months in temperate regions. The average daytime concentrations of surface O_3_ are largely dependent on the primary air pollutant levels within the region, ranging from 10 parts per billion (ppb) over the Pacific Ocean to more than 100 ppb down‐wind of large industrial emitting sources (Myhre *et al*. 2013). Precursors can travel far from the source on the regional, intercontinental and hemispheric scale before reacting (Cooper & Derwent 2013). Increasing O_3_ precursors in East Asia have been identified as major drivers of rising spring and summer background [O_3_] in the western United States over the past decades, despite control of local emissions (Cooper *et al*. 2010; Lin *et al*. 2017).

Past trends and future estimates of surface [O_3_] depend on legislation and control of precursor emissions in large part because O_3_ is a short‐lived molecule, lasting for only 22–24 days in the troposphere (Young *et al*. 2013; Hu *et al*. 2017). Thus, regulations can drastically improve O_3_ pollution in a short period of time. From 1990 to 2013, global mean [O_3_] increased by 8.9% (Brauer *et al*. 2016), but concentrations decreased in parts of the United States and Western Europe. The United States enacted more stringent NO_x_ regulations with the U.S. National Ambient Air Quality Standard (NAAQS) in 1997 (Environmental Protection Agency, 1997) and subsequently [O_3_] decreased over the past two decades (Brauer *et al*. 2016). In stark contrast, surface [O_3_] in East Asia increased by 25–50 ppb over the past 25 years, with a tripling of NO_x_ emissions over that time (Lin *et al*. 2017). Under the IPCC emissions scenarios, future [O_3_] will only decrease with the most optimistic, low emission scenario (RCP 2.6), and will either remain at current levels or increase under the other scenarios (Eyring *et al*. 2013). Future climate change could also increase the frequency of summer droughts and heatwaves, conditions that favour high [O_3_] in some regions (Meehl *et al*. 2018).

## Effects of Recent Increases in [CO_2_] and [O_3_] on Terrestrial Plant Productivity

The increase in atmospheric [CO_2_] along with the intensification of the global N cycle and extension of growing seasons with rising temperatures have enhanced global plant productivity (Fowler *et al*. 2013; Zhu *et al*. 2016; Peñuelas *et al*. 2017). Evidence from long‐term flux tower records suggests that net ecosystem production in Northern Hemisphere forests has increased over the past three decades (Keenan *et al*. 2013; Smith *et al*. 2016; Fernández‐Martinez *et al*. 2017), and satellite observations have reported widespread greening of vegetated land areas across the globe (Zhu *et al*. 2016). Greater productivity of Northern Hemisphere forests and agricultural lands in recent decades is thought to have contributed to increased seasonal photosynthetic drawdown of atmospheric [CO_2_], which has increased by ~50% at 45° N since the 1960s (Graven *et al*. 2013; Gray *et al*. 2014; Zeng *et al*. 2014; Forkel *et al*. 2016). Ecosystem models have been used to attribute the relative importance of rising [CO_2_], nitrogen deposition, climate change and land‐use change to the greening trend, and highlight the important role that CO_2_ fertilisation effects on vegetation (*i.e*. increased photosynthesis and water use efficiency) have played on a global scale (*e.g*. Devaraju *et al*. 2016; Forkel *et al*. 2016; Zhu *et al*. 2016; Keeling *et al*. 2017; Piao *et al*. 2018). By compiling data from inventory, simulation and atmospheric studies, Schimel *et al*. (2015) estimated that the CO_2_ fertilisation effect on the terrestrial biosphere potentially absorbed up to 30% of CO_2_ emissions from 1990 to 2007. McGrath & Lobell (2011) used historical yield and climate records to estimate that CO_2_ fertilisation increased maize and soybean yields by 9% and 14%, respectively, over the past 50 years. However, tree ring and isotope analysis studies of primarily Northern Hemisphere trees have found less evidence of CO_2_ fertilisation effects (Gedalof & Berg 2010), and instead suggest that other processes including forest regrowth after land abandonment or woody encroachment may be more important contributors to the enhanced terrestrial C sink (Peñuelas *et al*. 2011). More research is needed to understand the role of CO_2_ fertilisation on vegetation in the global C cycle, which strongly influences future atmospheric [CO_2_]. Unfortunately, there is also recent evidence that the land C sink is weakening, and that CO_2_ and N fertilisation effects on plant physiological processes may slow in the future as the negative effects of warmer temperatures and extreme droughts dominate ecosystem productivity (Peñuelas *et al*. 2017). The hypothesis that the terrestrial biosphere is currently at a turning point, shifting from a period when CO_2_ and N fertilisation dominate the global C cycle to a period when warming and drought stress dominate, highlights the immediate need for further understanding of the combined impacts of rising [CO_2_], [O_3_], temperature and drought stress on plant physiology and ecology, which can only be gained through multi‐factor experimentation.

The effects of increasing [O_3_] on global plant productivity over the past 150 years have received less attention than rising [CO_2_], in part because of the complexity of plant responses to O_3_ pollution, and in part because understanding is less complete. Mills *et al*. (2018) recently synthesised global O_3_ monitoring data, and estimated O_3_ risk for different vegetation types based on their growing seasons. There is a paucity of O_3_ monitoring information from rural sites in the Southern Hemisphere where concentrations tend to be lower than in the Northern Hemisphere. In North America, Europe and Asia critical limits for damage to vegetation are exceeded in many regions (Mills *et al*. 2018). The deposition of O_3_ on the terrestrial surface is highly seasonal and varies with land cover (Hardacre *et al*. 2015). Grasslands, agricultural lands and deciduous forests tend to show larger O_3_ fluxes and annual deposition rates than tundra and coniferous forests (Hardacre *et al*. 2015). Oliver *et al*. (2018) examined the impact that increased concentrations of O_3_ have had on European vegetation at the regional scale using a land surface model. Their simulations suggest that rising [O_3_] from 1901 to 2001 decreased European gross primary productivity by 3–9% on average, with some regions showing up to 30% decreases in gross primary productivity. Using a fully coupled chemistry–carbon–climate model, Yue *et al*. (2017) estimated that current [O_3_] in China is reducing annual net primary productivity by ~10.1–17.8%, and using historical ground‐level monitoring data, McGrath *et al*. (2015) estimated that O_3_ pollution reduced U.S. soybean and maize yields by 5–10% over the past 30 years. Tai & Martin (2017) used a partial derivative‐linear modelling approach, and also concluded that O_3_ pollution has had a significant negative impact on wheat, maize and soybean production in North America and Europe. However, other estimates of the effects of [O_3_] based on long‐term monitoring of background concentrations suggest much more limited effects, for example in European forests (Cailleret *et al*. 2018). The dynamic temporal and spatial nature of O_3_ pollution and the lack of long‐term data have limited global assessments of the impact of O_3_ on terrestrial ecosystems, but synthesis of crop responses to O_3_ pollution suggests that O_3_ constrains crop yields to a similar degree as nutrient, heat and aridity stress (Mills *et al*. 2018). Thus, greater attention to O_3_ pollution and its impact on terrestrial productivity is needed.

## Response of Plant Productivity to Future [CO_2_] and [O_3_]

Understanding of plant responses to rising atmospheric [CO_2_] and [O_3_] is fundamental to predictions of future atmospheric composition and global climate, global net primary productivity and ecosystem service provisioning, and remains one of the largest uncertainties in global terrestrial models. For nearly 30 years, the effects of elevated [CO_2_] and more recently [O_3_] on plant productivity have been experimentally studied at Free Air CO_2_ Enrichment (FACE) facilities (Koike *et al*. 2015; Kimball 2016; Norby *et al*. 2016). These facilities allow for increased concentration of CO_2_ and O_3_ in relatively large experimental plots without enclosures, and enable ecosystem‐scale experimentation, which can inform terrestrial ecosystem models (*e.g*. Walker *et al*. 2015). Although the first FACE experiments were predominantly sited in temperate regions, leaving a gap in our understanding of tropical and boreal system responses to rising [CO_2_] (Leakey *et al*. 2012), new FACE experiments in different biomes are beginning to address that gap (*e.g*. Tausz‐Posch *et al*. 2012; Bourgault *et al*. 2016; Britto de Assis Prado *et al*. 2016; DaMatta *et al*. 2016; Ellsworth *et al*. 2017; Rakocevic *et al*. 2018). Still, there are no large‐scale FACE experiments on the whole of the African continent.

The multi‐year duration of many FACE experiments as well as the size of individual FACE rings (up to 30 m in diameter) and ability to accommodate interactive treatments has improved understanding of the interactions of rising [CO_2_] with nutrient availability, rising temperatures, heat stress and variation in precipitation and plant water availability (*e.g*. McCarthy *et al*. 2010; Norby *et al*. 2010; Reich *et al*. 2014; Obermeier *et al*. 2017; Uddin *et al*. 2018; Gray *et al*. 2016; Norby *et al*. 2016). Translating this knowledge into global vegetation models is still a challenge, but some key discoveries are emerging. For example, the importance of nutrient availability in determining ecosystem responses to elevated [CO_2_] has long been recognised (*e.g*. Hungate *et al*. 2003; Ainsworth & Long 2005), necessitating the coupling of C and N cycles in models in order to accurately predict CO_2_ response. But the degree to which N limitation has dampened CO_2_ fertilisation has varied among FACE experiments. Recently Terrer *et al*. (2018) used an economics framework to address this variation and synthesise FACE experiments in forests and grasslands. They defined a metric termed ‘N acquisition return on investment’ as the relative increase in N acquisition divided by the relative increase in belowground C allocation, and then related the CO_2_ fertilisation effect on aboveground biomass to the N acquisition metric. In nutrient‐limited ecosystems, the relative efficiency of plant N acquisition, which was influenced by mycorrhizal association and N fixation capacity, in part determined the biomass response to elevated [CO_2_] (Terrer *et al*. 2018). They reported a continuum of CO_2_ fertilisation across plants and systems, with ectomycorrhizal‐associated plants at one end showing strong CO_2_ fertilisation and plants with arbuscular mycorrhizal associations showing no or weak growth responses to elevated [CO_2_]. This study provides a new framework for modelling the interaction of rising [CO_2_] and N acquisition.

The importance of nutrient supply, water availability and temperature in constraining biomass response to elevated [CO_2_] has been emphasised in recent studies of experiments in grassland ecosystems. At the biodiversity, [CO_2_] and N (BioCON) experiment on a perennial grassland in Minnesota, USA, elevated [CO_2_] failed to stimulate total biomass of plants when water supply was reduced by ~40% and N supply was limiting. However, if N, water or both were provided at ambient or elevated levels, then elevated [CO_2_] stimulated biomass by ~30% (Reich *et al*. 2014). In a C_3_ grassland in Germany, stimulation of aboveground biomass at elevated [CO_2_] was strongest when rainfall and temperature were average, and CO_2_ fertilisation of biomass was reduced or absent under extremely high or low soil moisture and/or temperature (Obermeier *et al*. 2017). In a native grassland in southeast Tasmania, seasonal rainfall patterns, not total annual rainfall, determined response to elevated [CO_2_] (Hovenden *et al*. 2014). There, high summer rainfall was associated with strong biomass stimulation at elevated [CO_2_], while high spring or autumn rainfall had the opposite effect. These important interactions were only discovered because of long‐term experimentation at FACE facilities, something that has not occurred to the same degree with elevated [O_3_] experiments. This gap in long‐term experimentation with elevated O_3_ may be one of the reasons that the interactive effects of O_3_, CO_2_, drought and increasing temperature are less clear (Emberson *et al*. 2018).

The FACE experiments in cropping systems have quantified the effects of elevated [CO_2_] or [O_3_] on crop yield quantity (Long *et al*. 2005, 2006) and quality (Yang *et al*. 2007; Myers *et al*. 2014; Kou *et al*. 2017), tested interactions with nutrient supply (Markelz *et al*. 2011; Weigel & Manderscheid 2012; Manderscheid *et al*. 2018), drought (Erbs *et al*. 2012; Manderscheid *et al*. 2014; O'Leary *et al*. 2015; Gray *et al*. 2016) and warming or variation in seasonal temperature (Hasegawa *et al*. 2013; Ruiz‐Vera *et al*. 2013; Bishop *et al*. 2014; Cai *et al*. 2016; Wang *et al*. 2018). An understanding of agricultural ecosystem responses to rising [CO_2_] and [O_3_] is important not only from a food security perspective, but also because agriculture contributes significantly to global net primary productivity (Gray *et al*. 2014; Zeng *et al*. 2014). Results from recent crop FACE experiments have reversed the long‐held hypothesis about how drought and rising [CO_2_] interact (Fitzgerald *et al*. 2016; Gray *et al*. 2016; Houshmandfar *et al*. 2016). In both wheat and soybean, yield gains at elevated [CO_2_] were higher in wet growing seasons as opposed to dry growing seasons, and higher early season biomass accumulation at elevated [CO_2_] had increasingly negative consequences when late‐season conditions were very dry (Fitzgerald *et al*. 2016; Houshmandfar *et al*. 2016). In these crops, early season stimulation of aboveground biomass at elevated [CO_2_] in some years produced a large canopy with high water demand. Even though elevated [CO_2_] reduced stomatal conductance at the leaf level, canopy size counteracted that response, and increased water demand of a larger canopy had a negative impact on seed yield during late‐season drought stress (Gray *et al*. 2016; Houshmandfar *et al*. 2016). Decreased transpiration at elevated [CO_2_] is one of the mechanisms that contributes to generally lower nutrient (*e.g*. protein, iron, zinc) content in the grains of crops exposed to elevated [CO_2_] (Myers *et al*. 2014). While many studies have shown a reduction in mineral nutrition in crops grown at elevated [CO_2_], recent studies that investigated the combination of rising [CO_2_] and warming reported that elevated temperatures can restore mineral concentrations to ambient conditions in soybean (Köhler *et al*. 2019; Palacios *et al*. 2019). Whether other crops will also show this general trend remains to be tested, as does the impact of the interactions of rising [O_3_], rising [CO_2_] and climate change on yield quality.

The FACE experiments are now moving towards experimentation aimed at adaptation of cropping and forest systems to future climates by harnessing genetic variation and transgenic approaches (*e.g*. Aspinwall *et al*. 2015; Hiraoka *et al*. 2017; Köhler *et al*. 2017). Recent FACE experiments with silviculture species have reported that genotypic variation in dry mass productivity is similar in ambient atmospheres as well as those with elevated [CO_2_] and [O_3_], implying that genotypes that are most productive today will continue to be most productive in a high [CO_2_], high [O_3_] future (Resco de Dios *et al*. 2016; Hiraoka *et al*. 2017). This is less clear for food crops, where inadvertent selection for CO_2_ responsiveness or O_3_ tolerance has not been found (Ainsworth *et al*. 2008). However, the large size of FACE rings along with spatially‐relevant statistical designs can enable screening potentially hundreds of crop genotypes at elevated [CO_2_] and/or [O_3_] (Yendrek *et al*. 2017b), and therein identifying promising lines. While screening genetic populations in the field under elevated [CO_2_] and/or [O_3_] along with higher temperature and reduced water availability is not yet feasible, long‐term studies have enabled identification of genotypes that are consistently CO_2_‐responsive or O_3_‐tolerant across variable growing seasons (*e.g*. Sanz‐Sáez *et al*. 2017; Yendrek *et al*. 2017a). Given that recent models have projected significant decreases in yields of major crops by the end of the 21st century, even with CO_2_ fertilisation effects (*e.g*. Hsiang *et al*. 2017), investment in such long‐term research is critical to meeting future demand for food and fuel.

## Conclusion

The global CO_2_ and O_3_ cycles have greatly intensified over the past 100 years. Today, terrestrial ecosystems take up nearly one‐third of the CO_2_ emitted from anthropogenic sources and one‐fifth of the tropospheric O_3_ produced by chemical production. In recent decades, rising CO_2_ has likely contributed to the greening of the planet, while O_3_ pollution has likely reduced terrestrial net primary productivity. The capacity of ecosystems to continue to filter atmospheric pollutants has important consequences for future climate and provisioning of ecosystem goods and services. However, recent studies suggest that the ability for terrestrial ecosystems to buffer anthropogenic emissions will diminish with rising temperature and drought stress. Thus, it is critical that we develop deeper understanding of the effects of rising [CO_2_] and [O_3_] pollution on net primary productivity, ecosystem C storage and crop production in agro‐ecosystems, and the interactive effects of rising temperature, variable water supply and nutrient availability.
